# Infective endocarditis: association between origin of causing bacteria and findings during oral infection screening

**DOI:** 10.1186/s12903-022-02509-3

**Published:** 2022-11-15

**Authors:** Therese Thoresen, Stina Jordal, Stein- Atle Lie, Friederike Wünsche, Martha Rolland Jacobsen, Bodil Lund

**Affiliations:** 1 The Department of Clinical Dentistry, University of Bergen, Bergen, Norway; 2 Department of Oral and Maxillofacial Surgery, The Head and Neck Clinic, Haukeland Hospital, Bergen, Norway; 3 Department of Medicine, Section of Infectious Disease, Haukeland University Hospital, Bergen, Norway; 4 Department of Dental Medicine, Division of Oral Diagnostics and Rehabilitation, Karolinska Institutet, Alfred Nobels Allé 8, 141 52 Stockholm, Huddinge Sweden; 5 Department for Oral and Maxillofacial Surgery and Jaw Orthopedics, Medical Unit of Plastic Surgery and Oral and Maxillofacial Surgery, Karolinska University Hospital, Stockholm, Sweden

**Keywords:** Infective endocarditis, Oral health, Viridans streptococci, Oral infection screening

## Abstract

**Background:**

Oral streptococci represent the causing microorganism for infective endocarditis (IE) in many patients. The impact of oral infections is questioned, and it has been suggested that bacteraemia due to daily routines may play a bigger part in the aetiology of IE. The aim of this study was to examine the association between oral health and infective endocarditis caused by oral bacteria in comparison with bacteria of other origin than the oral cavity.

**Methods:**

A retrospective study was conducted at Haukeland University Hospital from 2006- 2015. All consecutive adult patients admitted to hospital for treatment of IE and subjected to an oral focus screening including orthopantomogram, were included. The clinical, radiological and laboratory characteristics of the patients, collected during oral infectious focus screening, were analysed. Patient survival was calculated using Kaplan–Meier and mortality rates were compared using Cox-regression.

**Results:**

A total of 208 patients were included, 77% (*n* = 161) male patients and 23% (*n* = 47) female, mean age was 58 years. A total of 67 (32%) had IE caused by viridans streptococci. No statistically significant correlation could be found between signs of oral infection and IE caused by viridans streptococci. The overall mortality at 30 days was 4.3% (95% CI: 1.6–7.0). There was no statistical difference in mortality between IE caused by viridans streptococci or *S. aureus* (HRR = 1.16, 95% CI: 0.57–2.37, *p* = 0.680).

**Conclusion:**

The study indicates that the association between origin of the IE causing bacteria and findings during oral infection screening might be uncertain and may suggest that the benefit of screening and elimination of oral infections in patients admitted with IE might be overestimated. However, the results should be interpreted with caution and further studies are needed before any definite conclusions can be drawn.

## Introduction

Infective endocarditis (IE) defined as an infection of the heart valves or endocardium, displays high morbidity and mortality rates despite improved diagnostics and treatment. IE is most commonly in declining order caused by *Staphylococcus aureus*, viridans streptococci followed by enterococci [1]. Less frequent causes are coagulase-negative Staphylococci (CoNS) and bacteria of the HACEK-group, including *Haemophilus* spp., *Aggregatibacter* spp*., Cardiobacterium hominis, Eikenella corrodens,* and *Kingella kingae* [2, 3]. The bacteria manifests as a biofilm that may cause valve dysfunction and insufficiency, aneurysm or more rarely fistulas. Since oral viridans streptococci are the second most common cause of IE, the oral infection screening and subsequent elimination of suspected infectious foci have traditionally been considered an important component of the treatment protocol [4].

Predisposing factors for IE are prosthetic heart valves or cardiac devices, valvular or congenital heart diseases, intravenous drug use (IVDU), previous episode of IE, and immunosuppression [3]. Also, recent dental or surgical procedures have been suggested to increase the risk for IE in risk patients [5]. However, up to 40% of patients diagnosed with IE lacks previously known risk factors [6].

The clinical presentation of IE varies substantially and is often non-specific [5]. IE caused by *S. aureus* typically presents as a severe infection with peripheral septic embolization whereas IE caused by oral bacteria most often display a subacute course, where symptoms may last for weeks to months before diagnosed. The mortality rates are higher for IE caused by *S. aureus* than for viridans streptococci [2]*.* Nowadays the acute and subacute division are historical and instead description of the valve involved, source of infection, and native or prosthetic valves are considered more relevant since it has implications for the choice of treatment [7].

By leaking through the oral mucosal barrier, oral bacteria can enter the bloodstream and thus reach the endocardium. The viridans streptococci, as well as *S. aureus* and enterococci, carry the surface adhesins MSCRAMMS (microbial surface component reacting with adhesive matrix molecules) that mediate adhesion to damaged heart valves and vegetations [8]. In viridans streptococci the enzymes glucosyltransferase and fructosyltransferase produces the surface polysaccharide glucan from dietary sugars. Although probably not a prerequisite for development of IE, this molecule increases the bacterial adhesion to fibrin-plated clots and damaged endothelium of the heart valves. The virulence factor FimA seen in some viridans streptococci is also suggested to have importance in adherence and subsequent vegetation leading to IE [9]. Invasive dental procedures, such as tooth extraction, oral surgery or subgingival periodontal treatment allow oral bacteria to shed into the bloodstream. Bacteraemia may also occur during normal daily activities such as toothbrushing, flossing, and chewing [10].

Patients with IE are always hospitalized and treated with long-term intravenous antimicrobials alone or in combination with cardiac surgery [11]. During the first days of hospitalization possible infection foci other than the heart valves, should be identified and eliminated. As part of this, patients admitted for IE should routinely be referred to a dentist for investigation and elimination of potential oral infectious foci [12]. In most countries, including Norway, national guidelines recommend that patients with increased risk of IE should be prescribed antibiotic prophylaxis before invasive dental procedures [13]. The rational is that the antibiotic administered just before the procedure, reduces/shortens bacteraemia and thereby suggestively decrease the risk of IE. The efficacy of antibiotic prophylaxis for prevention of IE remains a debated area and solid evidence of its efficacy is still lacking. Another proposed preventive measure is maintaining good oral status to reduce the oral bacterial load and the subsequent bacteraemia [14, 15]. Neglected oral hygiene and following periodontal disease are thought to increase the likelihood of bacteraemia in connection to normal daily activities such as chewing and toothbrushing [16].

To date the association between oral status and occurrence of IE caused by oral microflora is not definitely confirmed. Furthermore, the efficacy and benefit of the oral screening and infection elimination in IE patients is not known. The aim of this study was to evaluate the association between oral health and infective endocarditis caused by oral bacteria in comparison with bacteria of other origin than the oral cavity.

## Material and methods

### Study design and patient population

The study was designed as a retrospective observational study based on data from patients diagnosed with IE in the period 2006–2015 at Haukeland University Hospital (HUH), Bergen, Norway. Inclusion criteria were patients admitted to hospital for treatment of IE, diagnosis verified according to the modified Duke criteria [17], age above 18, and performed oral focus screening including orthopantomogram (OPG) defined as a panoramic single conventional x-ray image of the mandible, maxilla and surrounding structures. In brief, the main modified Dukes criteria includes blood culture positive for IE, echocardiogram positive for endocardial involvement, vegetation, or abscess formation of the endocardium and new or worsening valvular regurgitation [17]. Exclusion criteria were missing patient records at the oral and maxillofacial surgery department, missing OPG and previous admission to hospital for same episode of IE. In case of repeated admissions during the same year, only the first episode of IE was registered. The hypothesis was that there would be an association between poor oral health status and IE caused by viridans streptococci. Because of this observational design and lack of previous data a Power calculation was not performed. The study was approved by the regional committee for ethics in medical research (REC West, approval no. 2015/1170).

### Data management

The data was collected from the following sources: 1) patient records from the department Oral and maxillofacial surgery (OMS); 2) radiographic examination using OPG; 3) a local medical patient register of IE patients [18]. Information regarding the bacterial cause of IE was retrieved from the local IE register, in which all consecutive patients admitted to the hospital are registered. The microbiological data was based on results from culture of blood samples drawn at admission to the hospital. These samples were subjected to routine culturing at the department of microbiology at HUH. Two standardized case record forms were developed, one for examination of OPG and the other for evaluating the patient records. Data collection included the variables age, gender, last dental visit, subjective complaints regarding oral cavity, temporomandibular status, oral hygiene, caries, periodontal condition, wisdom teeth, maxillary sinus, number of teeth, dental apices status, number of endodontically treated teeth, condition of restorations, suspected infection foci, eventual bone pathology, suggested treatment and conducted treatment. Data regarding hospital admission, IVDU, presence of prosthetic heart valves, acquired or congenital heart valve defects, causing microorganisms, duration of symptoms at time of diagnosis, performed heart surgery during admission period, and mortality, were retrieved from the patient register. Data collection from the patients records from the OMS visits and OPG were performed blinded to all the information in the patient register, including the causing microorganism. Furthermore, during the evaluation of OPG the examiner did not have access to patient records and vice versa. Repeated calibration exercises were performed before and during onset of data collection both regarding examination of OPG and registration of data from patient records. Also, patient record review and OPG examination were done independently by two different people to prevent bias.

### Statistical analyses

Comparison of categorical variables were performed using Chi- square test and Fisher’s exact test. Survival probabilities were calculated using the Kaplan–Meier methods. Cox-regression models were used to compare mortality rates. Infective endocarditis according to Duke criteria was regarded as the independent variable. IBM SPSS PASW Statistics for Windows, Version 25.0 were used for statistical analysis. *P*-values less than 0.05 were considered statistically significant.

A *p* value ≤ 0.05 was considered statistically significant. IBM SPSS PASW Statistics for Windows, Version 25.0 were used for statistical analysis. Statistical calculations were performed using Chi- square test, Fisher’s test, and Kaplan–Meier analyses.

## Results

### Patient characteristics

A total of 306 patients were admitted to the hospital for treatment of IE during the study period. The number of patients fulfilling the stipulated inclusion was 208 patients, 77% (*n* = 161) male and 23% (*n* = 47) females, while the number of excluded patients was 98. The reason for exclusion in all cases were that the patient had been subjected to oral infection focus screening, but an OPG had not been taken. The mean annual incidence rate of IE per 100.000 inhabitants was 7.4 during the period. Clinical and microbiological characteristics of IE cases are shown in Table 1. There was a strong male predominance of the included patients, and the mean age was lower for men compared to women. The most frequent pathogen in declining order was staphylococci 33% (*n* = 70), divided into *S. aureus* 25% (*n* = 53) and the less common coagulase-negative staphylococci (CoNS) in 8% (*n* = 17). The second most common group were viridans streptococci 32% (*n *= 67). Of the total number of patients IVDU was the most common predisposing risk factor (*n* = 54), followed by previous episode of IE. The period of symptoms before diagnosed with IE was less than two weeks for 46% (*n* = 96) and more than two weeks for 54% (*n* = 112) of the patients. Information in the patient records regarding last dental treatment was missing for most of the cases. When stated, 12% (*n* = 25) had dental treatment less than three months ago and 5% (*n* = 11) had dental treatment more than three months ago.

**Table 1:** Descriptive data of 208 IE-patients admitted to Haukeland University Hospital from 2006–2015

**Variable**	**No (%)**
**Gender**	
Male	161 (77)
Female	47 (23)
**Age**	58 (SD: 18, Min/Max: 20/89)
Male	56 (SD: 18 Min/Max: 20/87)
Female	64 (SD: 19 Min/Max: 21/89)
**Type of bacteria**	
Viridans streptococci	67 (32)
Non-viridans streptococci	11 (5)
*S. aureus*	53 (25)
CoNS	17 (8)
Enterococci	30 (14)
HACEK	4 (2)
Other	17 (8)
No bacterial growth	13 (6)
**Mortality**	57 (27)
**Previous endocarditis**	27 (13)
**IVDU**	54 (27)
**Duration of symptoms**	
> 2 weeks	96 (46)
< 2 weeks	112 (54)
**Heart valve surgery**	
Elective	25 (12)
Acute	63 (30)
**Last dental treatment**	
< 3 months	25 (12)
> 3 months	11 (5)
Not stated	172 (83)
**Number of teeth**	
Edentolous	12 (5)
1–19	46 (22)
20–27	99 (48)
28–32	51 (25)
**Infectious foci according to patient record**	56 (27)
OPG	99 (48)

*Abbreviations*: *No* Number of patients, *CoNS* Coagulase-negative staphylococci, *HACEK-group Haemophilus* spp., *Aggregatibacter* spp., *Cariobacterium hominis*, *Eikenella corrodens,* and *Kingella kingae, IVDU* Intra venous drug user, *OPG* Orhtopanthomogram

### Mortality rates

The mortality at 30 days was 4.3% (95% CI: 1.6–7.0). At 1 year the mortality was 11.0% (95% CI: 6.7–15.3). The overall mortality at 5 was 29% (95% CI: 21–36.8), while it was 56.8% (95% CI: 41.3–72.2) at 10 years. Specified for viridans streptococci the mortality at 30 days was 3.0% (95% CI: 0–7.1) and for *S. aureus* 3.8% (95% CI: 0–9.1). At 1 year the mortality for viridans streptococci and *S. aureus* was 10.6% (95% CI: 3.2–18.0) and 12% (95% CI: 3.0–21.0) respectively. When performing Cox-regression no overall difference in mortality between *S. aureus* and viridans streptococci (HRR = 1.16, 95% CI: 0.57–2.37, *p* = 0.680) was found.

### Dental health status

The number of teeth was recorded as an index of previous and current oral health. One fourth of the patients had a full dentition of 28 or more teeth, while the remaining had a reduced dentition to different extent. Twelve patients were edentulous. From the dental records the treating surgeons concluded that there was a possible oral infection focus in 27% (*n* = 56) patients, while in the retrospective analyses of the OPG, the corresponding number was found to be 48% (*n* = 99). In five patients there were no comments in the patient records regarding the results of the oral infection screening (Table 1). Furthermore, the suggested dental treatment after screening was in several cases not fulfilled and, in some cases, the eventual need for treatment not commented at all, Table 2.

**Table 2:** Dental treatment performed in relation to suggested treatment plan

**Infectious foci elimination according to plan**	**No (%)**
Completed	7 (3)
Partially	47 (23)
No treatment performed	74 (36)
**No infectious foci registered by OMS**	75 (36)

*Abbreviations*: *No* Number of patients, *OMS* Oral and maxillofacial surgeons

According to the patient records 24% (*n* = 47) of the patients had no need for dental treatment. Extractions of teeth was the dominant suggestion of treatment 24% (*n* = 49), endodontic treatment was advocated for 2% (*n* = 4), while 22% (*n* = 46) needed a combination of different treatments. For the remaining 28% of the patients (*n* = 62) the recommended treatment was not specified or difficult to interpret.

From the dental records the surgeon had commented signs of periodontal disease in 19% (*n* = 39) of the patients, no periodontal disease for 12% (*n* = 25) and for 69% (*n* = 144) information about periodontal disease was missing. From the retrospectively investigated OPG 79% (*n* = 164) had signs of periodontal disease, while 21% (*n* = 44) had normal marginal bone levels. The difference between records from the clinical examination and the retrospective analyses regarding periodontal status was significant (*p* < 0.001).

### Association between oral health and causing microorganisms

Chi- square test showed that there were no statistically significant association between IE caused by viridans streptococci and suspected oral infection foci according to patient records or OPG. Furthermore, no association between IE caused by viridans streptococci and poor oral status regarding periodontal condition, apical infection, caries, or lesion in jawbone could be seen (Table 3).

**Table 3:** Different clinical findings in relation to IE caused by viridans streptococci. Total number of patients were 208. 67 patients had IE caused by viridans streptococci (Pearson chi- square 2- sided)

**Findings**	**No**	**Viridans + (%)**	***p*- value**
Lesions in jawbone	16	5/16 (31%)	0.932
Periodontal disease	164	50/164 (30%)	0.304
Apical radiolucency	116	32/116 (27%)	0.109
Caries	68	17/68 (25%)	0.121
Infection based on dental records	56	18/56 (32%)	0.922
Infection based on OPG	99	26/99 (26%)	0.080

*Abbreviation*: *No* Number of patients, *Viridans* + IE caused by viridans streptococci, *OPG* orthopanthomogram

### Association between oral health and survival

**Fig. 1:**
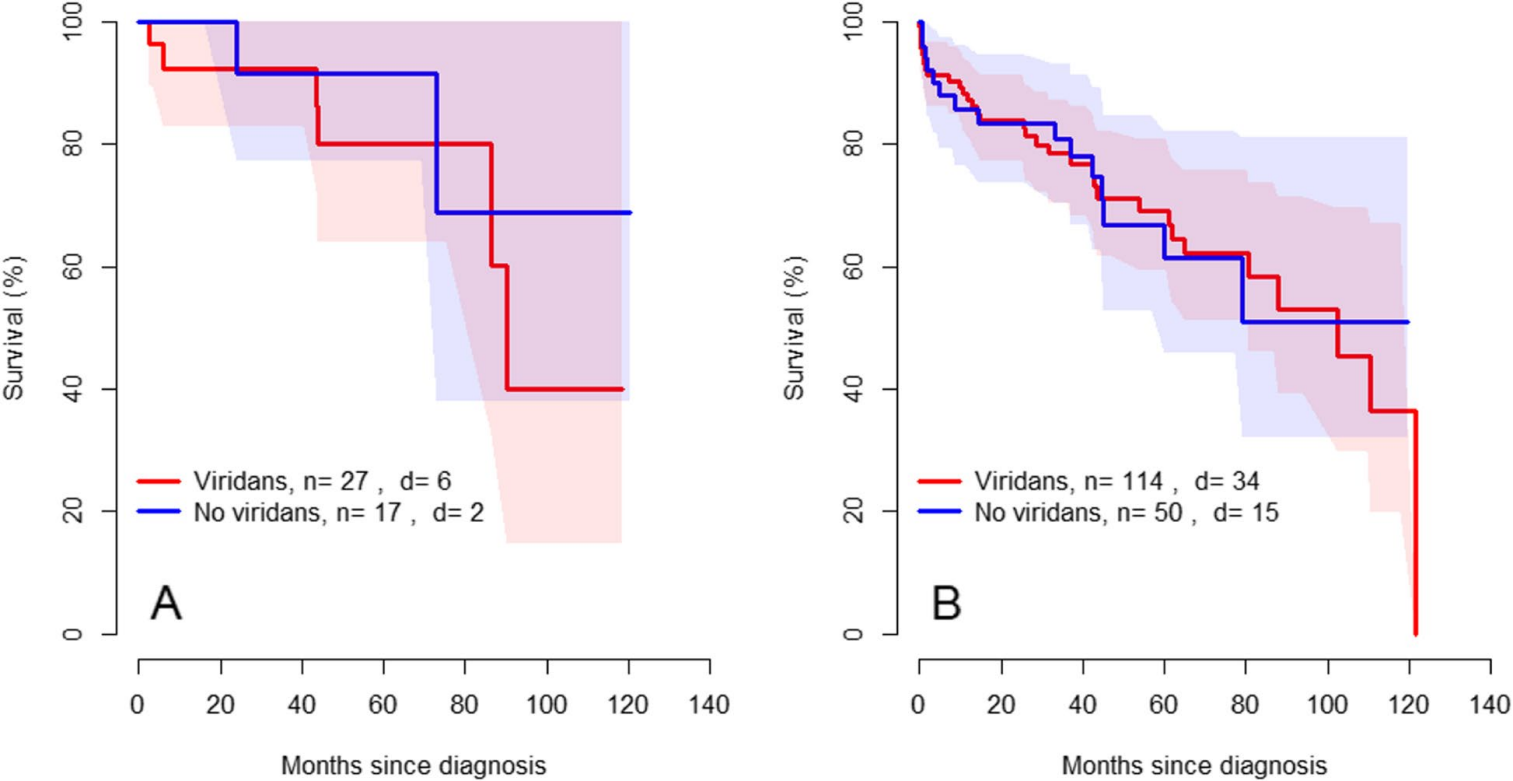
Kaplan- Meier curves showing mortality for patients with IE with 95% confidence interval. **A** Mortality for patients with IE without marginal bone loss. **B** Mortality for patients with IE with marginal bone loss

**Fig. 2:**
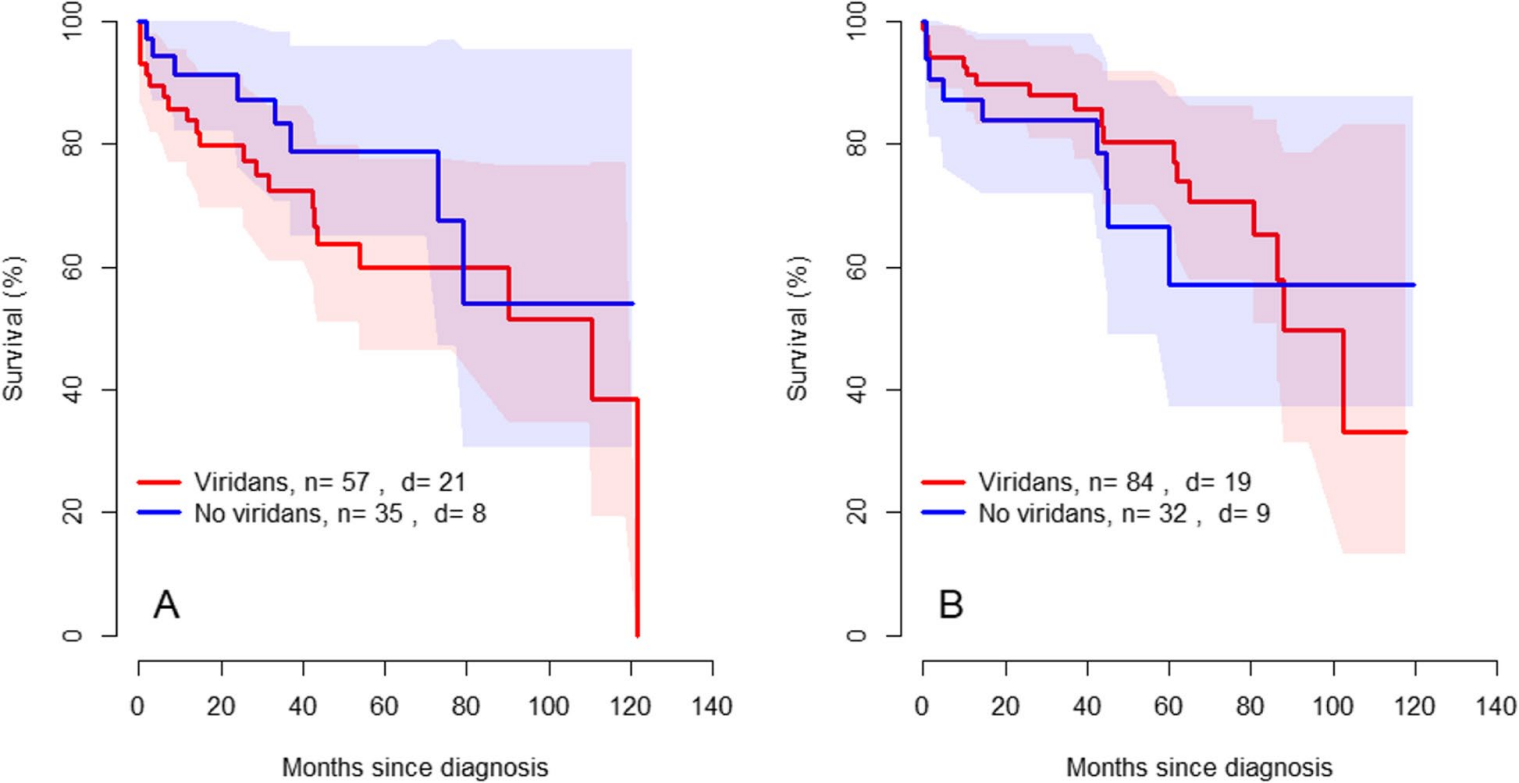
Kaplan- Meier curves showing mortality for patients with IE with 95% confidence interval. **A** Mortality for patients with IE without apical radiolucency on OPG. **B** Mortality for patients with IE with apical radiolucency on OPG

The risk of mortality was higher for patients with reduction of marginal bone height registered on OPG compared to patients with normal marginal status, independent of causing bacteria species. For patients with IE caused by viridans streptococci the risk of mortality was twice as high as compared to patients with IE caused by other bacteria when the marginal bone height was reduced according to OPG (Fig. 1). There was no risk difference noted for patients with radiological signs of apical infection, where the highest mortality rate could be seen in the group of patients with IE caused by viridans streptococci, but without apical signs of infection (Fig. 2).

## Discussion

To date it is a widely accepted approach to subject IE patients admitted to hospital to an oral infection focus screening and elimination as part of the treatment protocol [4, 12]. In fact, the rate of these screenings is sometimes used as a quality parameter [19]. Although these routines seize resources and are mutilating and costly for the patient, there is no solid scientific evidence to support these precautions. Until proven otherwise, screening is a reasonable approach because of the seriousness of IE and the indirect indication of link to oral status through causing microorganism [4]. However, considering the costs for the health care system, society and patients, the value and effectiveness of infection focus screening of IE patients should be investigated.

The current study could not confirm a significant association between oral infection foci and IE caused by viridans streptococci. In recent years, the association between invasive dental procedures causing bacteremia and subsequently giving rise to IE is vividly debated [10, 20]. An extension of this debate could be to question whether ongoing local oral infections are a risk factor, and if so, what type of conditions that are of relevance. In case of oral infection causing systemic symptoms, the uncertainty is less since this type of infection, by definition, causes shedding of oral bacteria into the bloodstream.

The study confirms high mortality rates for IE and are in range of other studies [1, 21, 22]. The high mortality rates might be explained by high prevalence of IVDU and comorbidities, but other explanatory factors cannot be excluded. The mortality rate for patients with IE caused by oral viridans group streptococci were similar as for the other bacteria. The results show that in patients with marginal bone loss the mortality was twice as high when IE was caused by viridans streptococci, compared to other bacteria. In contrast, there was no difference in mortality in patients with IE caused by viridans streptococci and presence of apical radiolucency. Earlier research shows that periodontal disease enhances bacteremia caused by viridans streptococci and has therefore been suggested to be a potential risk factor for IE [23]. Despite this, according to the dental records the impression was that the surgeons’ focus was periapical infection, rather than periodontitis. The higher mortality in the group with periodontal marginal bone loss suggests attention to, former or ongoing, periodontal disease when screening patients suffering from IE. An eventual association between marginal bone loss and mortality could either be due to a direct cause of IE or constitute a marker for reduced oral and/or general health [24]. However, these results must be interpreted with caution due to low number of patients and retrospective study design.

Timing of periodontal treatment is another issue worth discussing [25]. Acute periodontal treatment has the potential to worsen the bacteremia during hospital admission [26]. Other studies fail to show that postoperative course changes due to periodontal treatment [27]. Patients with periodontitis show larger tendency to poor oral hygiene, due to increased pocket depths and higher incidence of bleeding. Poor oral hygiene may also cause gingivitis which weaken the mucosal integrity, increasing the risk of bleeding and bacteremia due to normal activities. For this reason, poor oral hygiene can be regarded as a risk factor for IE since the normal infection defense barrier is weakened because of inflammation [16].

In 2007 ESC and NICE started revision of the antibiotic prophylaxis [28]. The arguments for changing the routines for antibiotic prophylaxis was lack of convincing scientific evidence for association between transient bacteremia and IE caused by invasive dental procedures in relation to the role of everyday routines [14]. Some studies show an association between invasive dental procedures and subsequent development of IE [29], while others fail to convincingly show such relationship [14]. Since our study did not reveal any association, we can to some extent support the studies suggesting that the daily routines, as chewing and toothbrushing, are a greater threat for individuals at risk for IE [10]. Today all antibiotic usage must be weighed against possible costs of resistance development. Consequently, because of weak association between invasive dental procedures and development of IE, some countries have changed their guidelines and refrain from, or decreased, the use of antibiotic prophylaxis for these patients [30, 31]. In the current study, the association to previous dental treatment preceding IE could not be evaluated due to frequent missing data. The fact that the surgeons refrain from including this in the patient record is noteworthy, considering the common notion of its importance. In Norway, the national guidelines stipulate that antibiotic prophylaxis should be given to all patients with high risk of IE [32]. Thus, it can be assumed that this precaution has been applied by the patients’ dentist in most cases. This could be one of the explanations for the lack of information regarding previous dental treatment in the patient records.

Suggested treatment by the dental surgeon was not executed for more than one third of the patients. Possible explanations for this can be due to either discharge from hospital or that the health status was too compromised to allow dental treatment. According to the patient records the focus was to eliminate apical infections. Due to lack of time before heart surgery and lack of equipment to perform endodontic procedures, the suggested treatment in several cases were extraction of teeth. Some suggest that dental treatment just before heart surgery increases the risk of mortality, while others claim that it makes no difference [25, 27]. Whether the postoperative outcome will differ between patients receiving dental treatment before cardiac valve surgery (CVS) or not are unconclusive according to a meta-analysis from 2019 [33].

The main limitation of this study is the retrospective design, which by nature makes collected data and analyses of missing data uncertain. Collecting data from patient records has an inherent component of interpretation due to non-aligned patient description. A prospective inclusion using a case record form and required study sample size would have resulted in more robust data collection and conclusions. To some extent address these shortcomings data collection was done independently by two researchers blinded to presence of IE caused by oral bacteria and preceded by repeated calibration exercises. Furthermore, data collection from OPG and patient records was done separately and blinded. However, in the absence of prospective studies on the subject a retrospective study can be justified and may serve as basis for hypothesis and design of future confirming studies of prospective design.

In summary, the results of the study indicate that there is no association between potential local oral infection foci and IE caused by oral bacteria. Considering the retrospective design of the study, and the seriousness of IE, these results should be interpreted with caution. However, considering the cost for both patients and the health care system, the results call for further studies, preferentially prospective, to verify these findings.

## Conclusions

The benefit of oral infection screening and elimination in patients with IE seems uncertain and there does not seem to be a straightforward association between ongoing dental infection and the development of IE caused by oral microorganisms. Furthermore, a risk hierarchy for different type of dental infections and the subsequent risk for IE could not be determined.

## Data Availability

The data that support the findings of this study were taken from a local register, but restrictions apply to the availability of these data, which were used under license for the current study, and so are not available publicly. Data are available from the corresponding author on reasonable request and with permission from SJ.

## Abbreviations

IE: Infective endocarditis
IVDU: Intravenous drug users
No: Number of patients
CoNS: Coagulase-negative staphylococci
HACEK-group: *Haemophilus* Spp., *Aggregatibacter* spp., *Cariobacterium hominis*, *Eikenella corrodens* and *Kingella kingae*
OPG: Orthophantomogram
HUH: Haukeland University Hospital
ESC: European Society of Cardiology
NICE: The National Institute for Health and Care Excellence
CVS: Cardiac valve surgery
